# Strategies for multi-case physics-informed neural networks for tube flows: a study using 2D flow scenarios

**DOI:** 10.1038/s41598-024-62117-9

**Published:** 2024-05-21

**Authors:** Hong Shen Wong, Wei Xuan Chan, Bing Huan Li, Choon Hwai Yap

**Affiliations:** 1 Department of Bioengineering, Imperial College London, Exhibition Road, London, SW7 2AZ UK; 2 Department of Chemical Engineering, Imperial College London, Exhibition Road, London, SW7 2AZ UK

**Keywords:** Physics informed neural network, Fluid flow, Deep learning, Hypernetworks, Biomedical engineering, Computational models, Machine learning

## Abstract

Fluid dynamics computations for tube-like geometries are crucial in biomedical evaluations of vascular and airways fluid dynamics. Physics-Informed Neural Networks (PINNs) have emerged as a promising alternative to traditional computational fluid dynamics (CFD) methods. However, vanilla PINNs often demand longer training times than conventional CFD methods for each specific flow scenario, limiting their widespread use. To address this, multi-case PINN approach has been proposed, where varied geometry cases are parameterized and pre-trained on the PINN. This allows for quick generation of flow results in unseen geometries. In this study, we compare three network architectures to optimize the multi-case PINN through experiments on a series of idealized 2D stenotic tube flows. The evaluated architectures include the ‘Mixed Network’, treating case parameters as additional dimensions in the vanilla PINN architecture; the “Hypernetwork”, incorporating case parameters into a side network that computes weights in the main PINN network; and the “Modes” network, where case parameters input into a side network contribute to the final output via an inner product, similar to DeepONet. Results confirm the viability of the multi-case parametric PINN approach, with the Modes network exhibiting superior performance in terms of accuracy, convergence efficiency, and computational speed. To further enhance the multi-case PINN, we explored two strategies. First, incorporating coordinate parameters relevant to tube geometry, such as distance to wall and centerline distance, as inputs to PINN, significantly enhanced accuracy and reduced computational burden. Second, the addition of extra loss terms, enforcing zero derivatives of existing physics constraints in the PINN (similar to gPINN), improved the performance of the Mixed Network and Hypernetwork, but not that of the Modes network. In conclusion, our work identified strategies crucial for future scaling up to 3D, wider geometry ranges, and additional flow conditions, ultimately aiming towards clinical utility.

## Introduction

The simulation of fluid dynamics in tube-like structures is a critical aspect of biomedical computational engineering, with significant applications in vascular and airway fluid dynamics. Understanding disease severity^1^, perfusion and transport physiology^2^, and the biomechanical stimuli leading to the initiation and progression of diseases relies on accurate fluid dynamics computations^3^. Traditionally, this involves extracting anatomic geometry from medical imaging and performing computational fluid dynamics simulations, but this process, although efficient, still demands computational time ranging from hours to days^4^. and the procedure is repeated for anatomically similar geometries, leading to an inefficient repetitive computational expenditure. Hastening fluid dynamics simulations to enable real-time results can enhance clinical adoption and potentially generate improvements in disease evaluation and decision-making.

In recent years, physics-informed neural networks (PINNs) have gained attention for approximating the behavior of complex, non-linear physical systems. These networks incorporate the underlying physics and governing equations of a system, allowing them to approximate solutions with good accuracy^5^. However, vanilla PINN requires individual training for each new simulation case, such as with variations in geometry, viscosity or flow boundary conditions, causing it to be more time-consuming than traditional fluid dynamics simulations.

Several past studies have provided strategies for resolving this limitation. Kashefi et al.^6^ proposed a physics-informed point-net to solve fluid dynamics PDEs that were trained on cases with varied geometry parameters, by incorporating latent variables calculated from point clouds representing various geometries. Ha et al.^7^ developed a hypernetwork architecture, where a fully connected network was used to compute weights of the original neural network, and showed that this could retain the good performance of various convolutional and recurrent neural networks while reducing learnable parameters and thus computational time. Felipe et al.^8^ developed the HyperPINN using a similar concept specifically for PINNs. Additionally, a few past studies have attempted to use parameterized geometry inputs in PINNs for solving fluid dynamics in tube-like structures of various geometries^9,10^, as demonstrated through 2D simulations.

In essence, by pre-training the PINN network for a variety of geometric and parametric cases (multi-case PINN), the network can be used to generate results quickly even for unseen cases, and can be much faster than traditional simulation approaches, where the transfer of results from one geometry to another is not possible. In developing the multi-case PINN, strategies and architectures proposed in the past for vanilla PINN are potentially useful. For example, Shazeer et al. used a “sparse hypernetwork” approach, where the hypernetwork supplies only a subset of the weights in the target network, thus achieving a significant reduction in memory and computational requirements without sacrificing performance^11^. A similar approach called DeepONet merges feature embeddings from two subnetworks, the branch and trunk nets, using an inner product^12,13^. Similar to hypernetworks, a second subnetwork in DeepONet can take specific case parameters as input, enhancing adaptability across diverse scenarios. Further, the gradient-enhanced PINN (gPINN) has previously been proposed to enhance performance with limited training samples, where additional loss functions imposed constraints on the gradient of the PDE residual loss terms with respect to the network inputs^14^.

However, the relative performance of various proposed networks for calculating fluid dynamics in tube-like structures is investigated here. We used a range of 2D tube-like geometries with a narrowing in the middle as our test case and investigated the comparative performance of three common PINN network designs for doing so, where geometric case parameters were (1) directly used as additional dimensions in the inputs to vanilla PINN (“Mixed Network”), (2) input via hypernetwork approach (“Hypernetwork”), or (3) inputs via partial hypernetwork similar to DeepONet (“Modes Network”).

To enhance the performance of multi-case tube flow PINN, we further tested two strategies. First, in solving fluid dynamics in tube-like structures, tube-specific parameters, such as distance along the tube centerline and distance from tube walls are extracted for inputs into the PINN network. This is likely to enhance outcomes as such parameters have a direct influence on fluid dynamics. For example, locations with small distance-to-wall coordinates require low-velocity magnitude solutions, due to the physics of the no-slip boundary conditions, where fluid velocities close to the walls must take on the velocities of the walls. Further, the pressure of the fluid should typically decrease with increasing distance along the tube coordinates, due to flow energy losses. Additionally, we investigated enhancing our multi-case PINN with gPINN^14^.

Our PINNs are conducted in 2D tube-like flow scenarios with a narrowing in the middle. As such, they are not ready for clinical usage, but they can be used to inform future work on 3D multi-case PINN with more realistic geometries and flow rates.

## Method

### Problem definition

In this study, we seek the steady-state incompressible flow solutions of a series of 2D tube-like channels with a narrowing in the middle, in the absence of body forces, where the geometric case parameter, ${\varvec{\lambda}}$, describes the geometric shape of the narrowing. The governing equations for this problem are as follows:

1 $$\nabla \cdot{\mathbf{u}}{ } = { }0,\quad {\varvec{x}} \in \user2{\Omega },\quad{ \lambda } \in {\mathbf{R}}^{{\varvec{n}}}$$

2 $$\left( {{\mathbf{u}}\cdot\nabla } \right){\mathbf{u}} = - \frac{1}{\rho }\nabla p + {\upnu }\nabla^{2} {\mathbf{u}}, {\varvec{x}} \in \user2{\Omega }, \user2{ \lambda } \in {\mathbf{R}}^{{\varvec{n}}}$$

3 $${\mathbf{u}}{ } = { }0,\quad {\text{at }}{\varvec{x}} = {{\varvec{\Gamma}}}_{{{\mathbf{wall}}}} \user2{ }$$

4 $$p = 0, \quad {\text{at }}{\varvec{x}} = {{\varvec{\Gamma}}}_{{{\mathbf{outlet}}}}$$

5 $$u = u_{max} *\left( {1 - \frac{{y^{2} }}{{R^{2} }}} \right), v = 0, \quad {\text{at}} {\varvec{x}} = {{\varvec{\Gamma}}}_{{{\mathbf{inlet}}}}$$

with fluid density $\rho$ = 1000 kg/m^3^, kinematic viscosity ${\upnu }$ = 1.85 m^3^/s, $p$ = $p\left( {\varvec{x}} \right)$ is the fluid pressure, ${\varvec{x}} = \left( {x,y} \right)$ is the spatial coordinates and ${\mathbf{u}}$ $=$ ${\mathbf{u}}$(${\varvec{x}},{\varvec{\lambda}}$) $=$ [$u$(${\varvec{x}},{\varvec{\lambda}}$), $v$(${\varvec{x}},{\varvec{\lambda}}$)]^T^ denotes the fluid velocity with components $u$ and $v$ in two dimensions across the fluid domain ${\varvec{\varOmega}}$ and the domain boundaries ${{\varvec{\Gamma}}}$. A parabolic velocity inlet profile is defined with $R$ as the radius of the inlet, and $u_{max} = 0.00925$ ms. prescribed. A zero-pressure condition is prescribed at the outlet. ${\varvec{\lambda}}$ is a $n$-dimensional parameter vector, consisting of two case parameters, ${\varvec{A}}$ and ${{\varvec{\upsigma}}}$, which describe the height (and thus severity) and length of the narrowing, respectively, given as:

6 $$R\left( x \right) = R_{0} - {\varvec{A}}e^{{ - \frac{{\left( {x - {\upmu }} \right)^{2} }}{{2{{\varvec{\upsigma}}}^{2} }}}}$$

where $R$ is the radius of the channel at a specific location, and ${R}_{0}$ and $\upmu$ are constants with values 0.05 m and 0.5 respectively. The Reynolds number of these flows is thus between 375 and 450.

### Network architecture

In this study, we utilize PINN to solve the above physical PDE system. Predictions of ${\varvec{u}}$ and $p$ are formulated as a constrained optimization problem and the network is trained (without labelled data) with the governing equations and given boundary conditions. The loss function ${\mathbf{\mathcal{L}}}\left( {\varvec{\theta}} \right)$ of the physics-constrained learning is formulated as,

7 $${\mathbf{\mathcal{L}}}\left( {\varvec{\theta}} \right) = \user2{ }\omega_{physics} {\mathbf{\mathcal{L}}}_{physics} + \omega_{bc} {\mathbf{\mathcal{L}}}_{BC}$$

$${\varvec{\theta}}^{\user2{*}} = \arg min_{W, b} \left( {{\mathbf{\mathcal{L}}}\left( {\varvec{\theta}} \right)} \right)$$

where ***W*** and ***b*** are weights and biases of the FCNN (see Eq. 11), ${\mathbf{\mathcal{L}}}_{physics}$ represents the loss function over the entire domain for the parameterized Continuity and Navier–Stokes equations, and ${\mathbf{\mathcal{L}}}_{BC}$ represents the boundary condition loss of the ${\varvec{u}}$ prediction. $\omega_{physics}$ and $\omega_{bc}$ are the weights parameters for the terms. A value of 1 is used for both as the loss terms are unit normalized. Loss terms can be expressed as:

8 $${\mathbf{\mathcal{L}}}_{physics} = \frac{1}{{V_{s}^{ - 2} {\varvec{N}}_{{{\varvec{domain}}}} }}\mathop \sum \limits_{{{\varvec{i}} = 1}}^{{\varvec{N}}} \left. {\left| {\nabla \cdot\hat{\user2{u}}} \right|^{2} } \right|_{{\varvec{\varOmega}}} + \frac{1 }{{\left( {V_{m} V_{s}^{ - 2} } \right)^{2} {\varvec{N}}_{{{\varvec{domain}}}} }}\mathop \sum \limits_{{{\varvec{i}} = 1}}^{{\varvec{N}}} \left. {\left| {\left( {\hat{\user2{u}}\cdot\nabla } \right)\hat{\user2{u}} + \frac{1}{\rho }\nabla \hat{p} - {\upnu }\nabla^{2} \hat{\user2{u}}} \right|^{2} } \right|_{{\varvec{\varOmega}}} \user2{ }_{\user2{ }}$$

9 $$\begin{aligned} {\mathcal{L}}_{BC} & = \frac{1}{{\left( {V_{m} V_{s}^{ - 1} } \right)^{2} {\varvec{N}}_{{{\varvec{wall}}}} }}\mathop \sum \limits_{{{\varvec{i}} = 1}}^{{\varvec{N}}} \left. {\left( {\hat{\user2{u}}} \right)^{2} } \right|_{{{{\varvec{\Gamma}}}_{{{\varvec{wall}}}} }} + \user2{ }\frac{1}{{\left( {V_{kg} V_{m}^{ - 1} V_{s}^{ - 2} } \right)^{2} {\varvec{N}}_{{{\varvec{outlet}}}} }}\mathop \sum \limits_{{{\varvec{i}} = 1}}^{{\varvec{N}}} \left. {\left( {\hat{p}} \right)^{2} } \right|_{{{{\varvec{\Gamma}}}_{{{\mathbf{outlet}}}} }} \\ & \;\; + \user2{ }\frac{1}{{\left( {V_{m} V_{s}^{ - 1} } \right)^{2} {\varvec{N}}_{{{\varvec{inlet}}}} }}\mathop \sum \limits_{{{\varvec{i}} = 1}}^{{\varvec{N}}} \left. {\left( {\hat{u} - u_{max} *\left( {1 - \frac{{y^{2} }}{{R_{inlet}^{2} }}} \right)} \right)^{2} } \right|_{{{{\varvec{\Gamma}}}_{{{\varvec{inlet}}}} }} \\ & \;\; + \user2{ }\frac{1}{{\left( {V_{m} V_{s}^{ - 1} } \right)^{2} {\varvec{N}}_{{{\varvec{inlet}}}} }}\mathop \sum \limits_{{{\varvec{i}} = 1}}^{{\varvec{N}}} \left. {\left( {\hat{v}} \right)^{2} } \right|_{{{{\varvec{\Gamma}}}_{{{\varvec{inlet}}}} }} \\ \end{aligned}$$

where ${\varvec{N}}$ is the number of randomly selected collocation points in the domain or at the boundaries, and *V*_*kg*_, *V*_*m*_ and *V*_*s*_ are the unit normalization of 1 kg, 0.1 m and 10.811 s respectively corresponding to the density $\rho$, inlet tube diameter 2$R_{0}$ and inlet maximum velocity $u_{max}$.

Training of the PINN was done using the Adam optimizer^15^, using a single GPU (NVIDIA Quadro RTX 5000). A feedforward fully connected neural network (FCNN), ${\varvec{f}}$, was employed in this work where the surrogate network model is built to approximate the solutions, $\widehat{{\user2{y }}} = \left[ {{\text{u}}\left( {{\varvec{x}},{\varvec{\lambda}}} \right),{\text{v}}\left( {{\varvec{x}},{\varvec{\lambda}}} \right),{\text{p}}\left( {{\varvec{x}},{\varvec{\lambda}}} \right)} \right]^{{\varvec{T}}}$. In the FCNN, the output from the network (a series of fully connected layers), $\widehat{{\user2{y }}}$(${\varvec{\psi}}_{\user2{ }} ;{\varvec{\theta}}$), where ${\varvec{\psi}}_{\user2{ }}$ represents the network inputs, was computed using trainable parameters ${\varvec{\theta}}$, consisting of the weights ${\varvec{W}}_{{\varvec{i}}}$ and biases ${\varvec{b}}_{{\varvec{i}}}$, of the $i$-th layer for $n$ layers, according to the equation:

10 $$\widehat{{\user2{y }}}\left( {{\varvec{\psi}}_{\user2{ }} ;{\varvec{\theta}}} \right) = {\varvec{W}}_{{\varvec{n}}} \left\{ {{\varvec{\varPhi}}_{{{\varvec{n}} - 1}} \circ{\varvec{\varPhi}}_{{{\varvec{n}} - 2}} \circ \ldots \circ{\varvec{\varPhi}}_{1} } \right\}\left( {{\varvec{\psi}}_{\user2{ }} } \right) + {\varvec{b}}_{{\varvec{n}}}$$

$${\varvec{\varPhi}}_{{\varvec{i}}} = \user2{ \alpha }({\varvec{W}}_{{\varvec{i}}} \left( {{\varvec{\varPhi}}_{{{\varvec{i}} - 1}} } \right) + {\varvec{b}}_{{\varvec{i}}} ),\quad for\, 2 < {\varvec{i}} < {\varvec{n}} - 1$$

where ${\varvec{\varPhi}}_{{\varvec{i}}}$ represents the nodes of the ${\varvec{i}}$th layer in the network. The Sigmoid Linear Unit (SiLu) function, ${\varvec{\alpha}}$, is used as the activation function and partial differential operators are computed using automatic differentiation^16^. All networks and losses were constructed using NVIDIA’s Modulus framework v22.09^17^, and codes are available at https://github.com/WeiXuanChan/ModulusVascularFlow.

### Mixed network, hypernetwork and modes network

**Figure 1:**
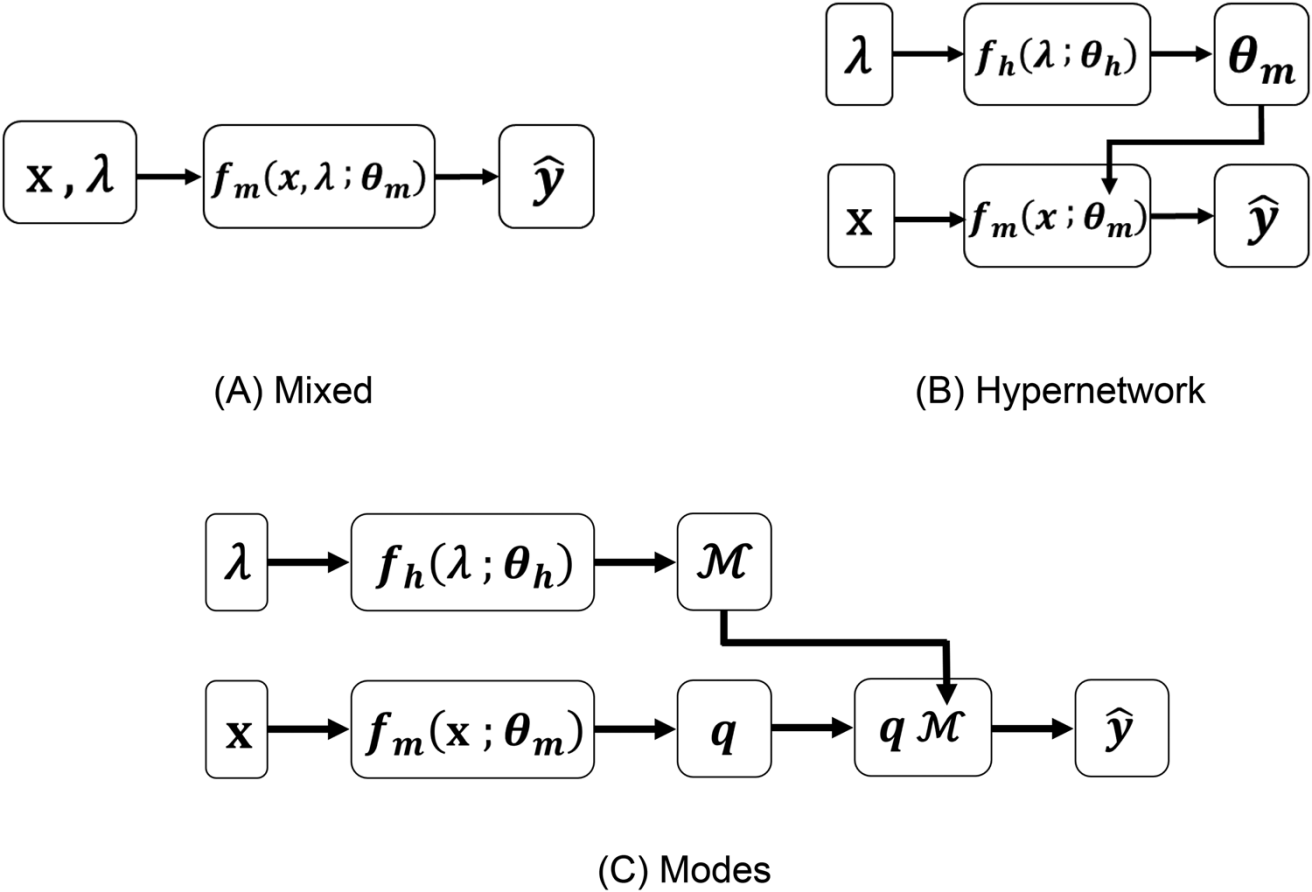
Schematic for the three different neural network architectures. (**A**) The “Mixed network” where the main network, ${\varvec{f}}_{{\varvec{m}}}$, takes in both coordinate parameters, **x**, and case (geometric) parameters, **λ**, to compute outputs variables, $\hat{\user2{y}}$, where hyperparameters, ${\varvec{\theta}}_{{\varvec{m}}}$, are optimized during training. (**B**) The “Hypernetwork” where ${\varvec{f}}_{{\varvec{m}}}$ is coupled to a side hypernetwork, ${\varvec{f}}_{{\varvec{h}}}$, which takes **λ** as inputs and outputs ${\varvec{\theta}}_{{\varvec{m}}}$ in ${\varvec{f}}_{{\varvec{m}}}$, and hyperparameters for the hypernetwork, ${\varvec{\theta}}_{{\varvec{h}}}$, are optimized during training. (**C**) The “Modes network” where ${\varvec{f}}_{{\varvec{h}}}$ outputs a modes layer, ${\text{M}}$, that is multiplied mode weights output by ${\varvec{f}}_{{\varvec{m}}}$, ${\varvec{q}}_{{}}$, to give output variable, $\hat{\user2{y}}$.

Three network architectures are investigated, as shown in Fig. 1. The number of learnable parameters in each NN architecture was kept approximately the same ($\pm 0.1\%$ difference) for comparison. In the Mixed Network approach, ${\varvec{\psi}}_{{}}$ consist of both ${\varvec{x}}$ and ${\varvec{\lambda}}$, and only one main FCNN network, ${\varvec{f}}_{{\varvec{m}}}$, is used to compute the velocity and pressure outputs.

11 $$\hat{\user2{y}} = {\varvec{f}}_{{\varvec{m}}} \left( {{\varvec{x}},{\varvec{\lambda}};{\varvec{\theta}}_{{\varvec{m}}} } \right)$$

In the Hypernetwork approach, ${\varvec{x}}$ is input into the main FCNN network, ${\varvec{f}}_{{\varvec{m}}}$, while ${\varvec{\lambda}}$ is input into a FCNN hypernetwork, ${\varvec{f}}_{{\varvec{h}}}$, which is used to compute the weights and biases (${\varvec{\theta}}_{{\varvec{m}}}$) of ${\varvec{f}}_{{\varvec{m}}}$. This can be mathematically expressed as:

12 $$\hat{\user2{y}} = {\varvec{f}}_{{\varvec{m}}} \left( {{\varvec{x}};{\varvec{\theta}}_{{\varvec{m}}} } \right)$$

$${\varvec{\theta}}_{{\varvec{m}}} = \user2{ f}_{{\varvec{h}}} \left( {{\varvec{\lambda}};{\varvec{\theta}}_{{\varvec{h}}} } \right)$$

where ${\varvec{\theta}}_{{\varvec{h}}}$ are the trainable parameters of ${\varvec{f}}_{{\varvec{h}}}$.

In the Modes Network, a hypernetwork, ${\varvec{f}}_{{\varvec{h}}}$, outputs a series of modes, **ℳ**. Its inner product with the main network (${\varvec{f}}_{{\varvec{m}}}$) outputs,*** q****,* is taken as the final output of the network to approximate flow velocities and pressures, expressed as:

13 $$\begin{aligned} {\mathbf{\mathcal{M}}} & = {\varvec{f}}_{{\varvec{h}}} \left( {{\varvec{\lambda}};{\varvec{\theta}}_{{\varvec{h}}} } \right) \\ {\varvec{q}} & = {\varvec{f}}_{{\varvec{m}}} \left( {{\varvec{x}};{\varvec{\theta}}_{{\varvec{m}}} } \right) \\ \hat{\user2{y}}_{{\varvec{i}}} & = \mathop \sum \limits_{{{\varvec{j}} = 1}}^{{\varvec{B}}} {\varvec{q}}_{{\varvec{j}}} {\mathbf{\mathcal{M}}}_{{{\varvec{ji}}}} \quad {\text{for }}i = 1,{ }2,{ }3 \\ \end{aligned}$$

where ${\varvec{\theta}}_{{\varvec{h}}}$ and ${\varvec{\theta}}_{{\varvec{m}}}$ are, again, the trainable parameters of ${\varvec{f}}_{{\varvec{h}}}$ and ${\varvec{f}}_{{\varvec{m}}}$, respectively. This formulation is previously proposed as the DeepONet^12,13^.

**Figure 2:**
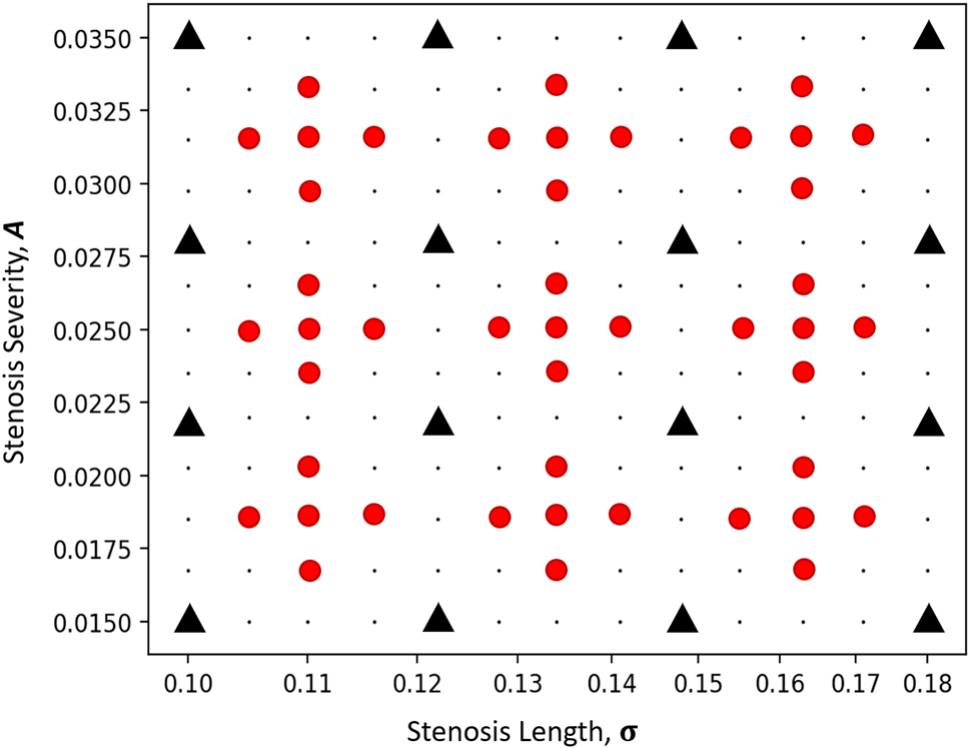
Illustration of the set of training and validation cases for multi-case training across a range of tube geometries with varying narrowness, ***A*** and narrowing length, ${{\varvec{\upsigma}}}$. Individual training cases are indicated by black “” and validation cases are indicated by red “”. The loss function is optimized with the training set and the accuracy of the network is evaluated with the validation set.

The NN architecture is trained for an arbitrary range of geometric parameters, ${\varvec{\lambda}} = \left\{ {{\varvec{A}},{{\varvec{\upsigma}}}} \right\}$, where ${\varvec{A}}$ varies between 0.015 and 0.035 and ${{\varvec{\upsigma}}}$ varies between 0.1 and 0.18. A total of 16 regularly spaced (***A***) and logarithmically spaced (${{\varvec{\upsigma}}}$) combinations are selected, and the performance of the three NN architectures is evaluated for the 16 training cases, as well as an additional 45 untrained cases. This is illustrated in Fig. 2.

A batch size of 1000 was employed, with 3840 batch points in each training iteration, resulting in a total of 3.8 million spatial points per epoch. The individual batch points used within each training step are compiled in Table 1.

**Table 1:** Batch points used per training iteration for each boundary condition imposed.

Region of interest	Boundary condition	Batch points
Inlet	Inlet profile (Eq. 5)	160
Outlet	Outlet pressure (Eq. 4)	160
Top wall	No-slip wall velocity (Eq. 3)	160
Bottom wall	No-slip wall velocity (Eq. 3)	160
Interior	Incompressible Navier–Stokes residuals	3200

### Computational fluid dynamics and error analysis

**Figure 3:**
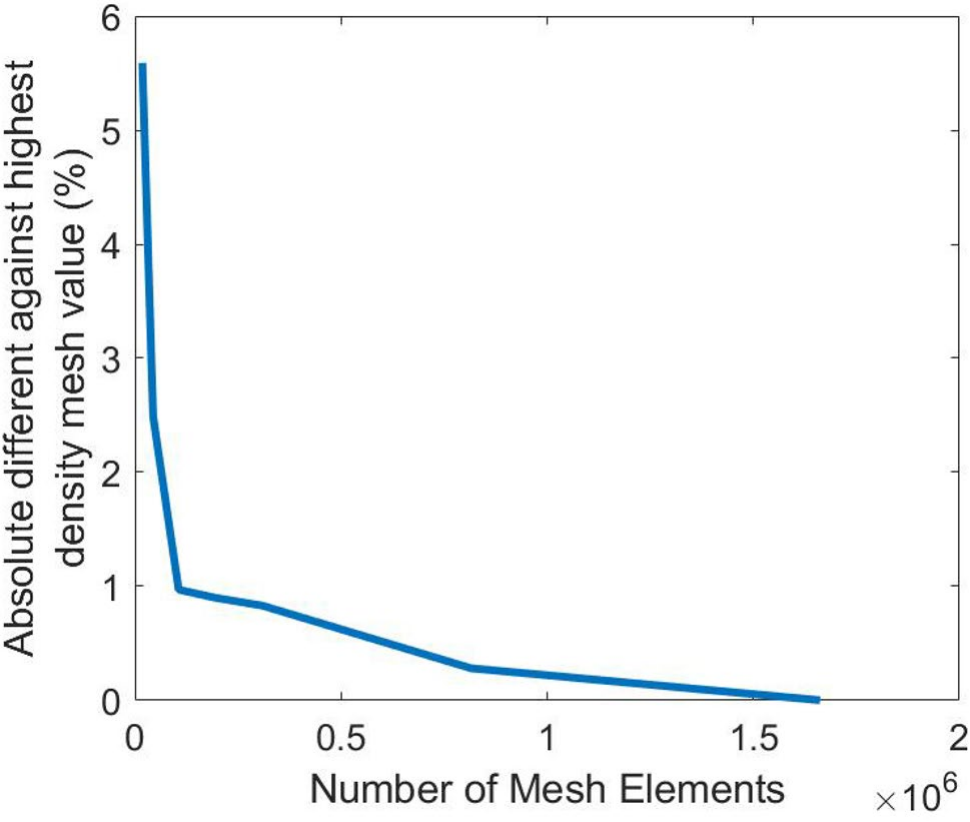
Maximum wall shear stress obtained for a 2D tube flow with narrowing (***A*** = 0.035 and **σ** = 0.10) across various mesh densities. The values are presented as a percentage difference relative to the highest tested density.

CFD ground truths of the training and prediction cases were generated using COMSOL Multiphysics v5.3 with the same boundary conditions set for the PINN. Mesh convergence was achieved by incrementally increasing the mesh size until the wall shear stress magnitude differed by approximately 0.5% compared to a finely resolved mesh, totalling around 1 million 2D triangular elements for each case model, shown in Fig. 3. Wall shear stress vector ($\overrightarrow {WSS}$) was calculated as:

14 $$\overrightarrow {WSS} = \mu \left| {\left( {\frac{{\nabla \vec{v} + \left( {\nabla \vec{v}} \right)^{T} }}{2}} \right)\hat{n}} \right|$$

where $\mu$ is the shear viscosity of the fluid, $\left( {\nabla \vec{v}} \right)$ is the gradient velocity tensor and $\hat{n}$ is the unit surface normal vector. The accuracy of the PINN was quantified using relative norm-2 error, $\varepsilon$, expressed as a percentage difference:

15 $$\varepsilon= \frac{{\sqrt {\mathop \sum \nolimits_{{{\varvec{i}} = 1}}^{{\varvec{N}}} \left| {{\varvec{y}}_{{{\text{PINN}}}} - {\varvec{y}}_{CFD} } \right|^{2} } }}{{\sqrt {\mathop \sum \nolimits_{{{\varvec{i}} = 1}}^{{\varvec{N}}} \left| {{\varvec{y}}_{CFD} } \right|^{2} } }} \times 100\%$$

This error was evaluated for the output variable ${\varvec{y}}$ on ${\varvec{N}}$ random collocation points.

### Tube-specific coordinate inputs, TSC

In the context of flows in a tube-like structure, we propose the inclusion of tube-specific coordinate parameters, referred to as TSCs. These additional variables, derived from the coordinates, are introduced as inputs into the PINN (as part of coordinate inputs, $x$, in Fig. 1, with the same resolution as $x$). 8 different TSCs were added: (1) “centerline distance”, $c = \left( { - 1,1} \right)$, which increases linearly along the centerline from the inlet to the outlet, (2) “normalized width”, $L_{n} = \left( { - 1,1} \right)$, which varies linearly across the channel width from the bottom and top wall, (3) $d_{sq} = 1 - L_{n}^{2}$, as well as multiplication combinations of the above variables, (4) $c^{2}$, (5) $L_{n}^{2}$, (6) $c \times d_{sq}$, (7) $c \times L_{n}$ and (8) $L_{n} \times d_{sq}$.

### Gradient-enhanced PINN, gPINN

In many clinical applications, obtaining patient-specific data is challenging, and the ability to train robustly with reduced cases would be beneficial. Therefore, we tested the use of gPINN, where the gradient of loss functions with respect to case inputs is added as additional loss functions for training. This aims to improve training robustness and reduce the number of training cases needed^14^. The addition of this derivative loss function is denoted as ${\mathbf{\mathcal{L}}}_{derivative}$, is hypothesized to enhance the sensitivity of the network to unseen cases close to the trained cases. This potentially allows for effective coverage of the entire case parameter space with fewer training cases. The approach involves additional loss functions:

16 $${\mathbf{\mathcal{L}}}\left( {{\varvec{W}},\user2{ b}} \right) = \user2{ }\omega_{physics} {\mathbf{\mathcal{L}}}_{physics} + \omega_{bc} {\mathbf{\mathcal{L}}}_{BC} + \omega_{derivative} {\mathbf{\mathcal{L}}}_{derivative}$$

17 $${\mathbf{\mathcal{L}}}_{derivative} = \frac{1}{{{\varvec{N}}_{{{\varvec{domain}}}} }}\mathop \sum \limits_{{{\varvec{i}} = 1}}^{{{\varvec{N}}_{{{\varvec{domain}}}} }} \left. {\frac{{d{\varvec{R}}_{{{\varvec{GE}}}} }}{d\lambda }} \right|_{{\varvec{\varOmega}}} + \user2{ }\frac{1}{{{\varvec{N}}_{{{\varvec{\Gamma}}}} }}\mathop \sum \limits_{{{\varvec{i}} = 1}}^{{{\varvec{N}}_{{{\varvec{\Gamma}}}} }} \left. {\frac{{d{\varvec{R}}_{{{\varvec{BC}}}} }}{d\lambda }} \right|_{{{\varvec{\Gamma}}}}$$

where $\omega_{derivative}$ is the weight parameter of the derivative loss function, ${\varvec{R}}_{{{\varvec{GE}}}}$ and ${\varvec{R}}_{{{\varvec{BC}}}}$ are the residual loss of the governing equation and boundary conditions, respectively, and ***N*** is the number of randomly selected collocation points in the domain.

## Results

### Advantages of tube-specific coordinate inputs

**Figure 4:**
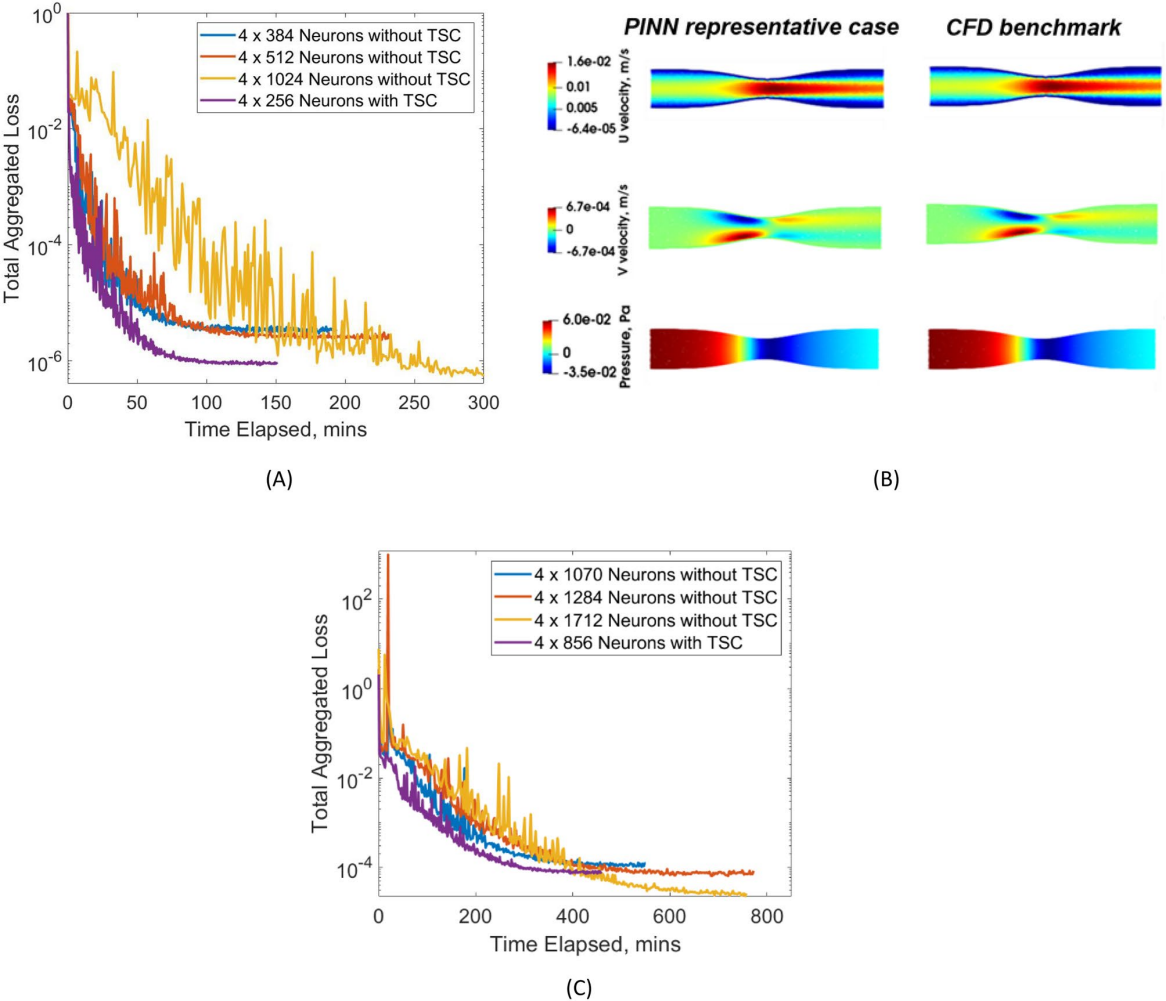
(**A**) Comparison of convergence for total aggregated loss plotted against time taken in minutes for training the narrowing case with ***A*** = 0.025 and **σ** = 0.134, using various neural network depth sizes as well as a smaller NN when employing “local coordinates inputs”. (**B**) Illustration of the flow results for single case training using 4 × 256 neurons with LCIs, show a good match between predictions from neural network and computational fluid dynamics (CFD) results. (**C**) Comparison of convergence for total aggregated loss plotted against time taken in minutes for multi-case training across various a range of narrowing severity, ***A*** and narrowing length, ${{\varvec{\upsigma}}}$, using the Mixed Network.(**A**) Single Case Training. (**B**) Fluid Flow – Single Case Training, 4 x 256 Neurons with LCI. (**C**) Multi-Case Training.

We first test the use of a vanilla FCNN on a single narrowing case, to assess the accuracy, sensitivity to network size, and utility of the TSC inputs. Results are shown in Table 2 and Fig. 4 for the single narrowing test case where ***A*** = 0.025, **σ** = 0.134. Figure 4A illustrates the successful convergence of the loss function during the training process, while Table 2 shows that, in comparison with CFD results, errors in velocities and errors are reasonably low. Figure 4B further demonstrates a visual similarity between network outputs and CFD simulation results. It should be noted that absolute errors in the y-direction velocity are not higher than those of other outputs, but as errors are normalized by the root-mean-square of the truth values, and because the truth flow field has very low y-direction velocities, the normalized y-direction velocity errors, $\varepsilon_{v}$, were higher. Accuracy and training can likely be enhanced with dynamic adjustment of weightage for the different loss functions and adaptive activation functions^18,19^, but such further optimizations are not explored here.

**Table 2:** Comparison of relative L2 error and computational expense for narrowing case with ***A*** = 0.025 and **σ** = 0.134, using various neural network depth size as well as a smaller NN when employing “local coordinates inputs”.

NN size [Layer x Neurons]	No. of hyperparameters	ε_u_	ε_v_	ε_p_	Approx. computational time (min)	GPU usage (GB)
4 × 384 without TSC	445,443	0.51%	2.63%	0.16%	135	1.87
4 × 512 without TSC	790,531	0.33%	2.26%	0.14%	150	2.36
4 × 1024 without TSC	3,153,923	0.18%	1.39%	0.11%	> 300	4.39
4 × 256 with TSC	200,707	0.36%	2.37%	0.12%	100	1.62

NN—neural network; TSC—tube-specific coordinate inputs; ε_u_, ε_v_, ε_p_—relative L2 error for U velocity, V velocity and pressure respectively.

Previous studies have reported that accurate results are more difficult without the use of hard boundary constraints^9^, where the PINN outputs are multiplied to fixed functions to enforce no-slip flow conditions at boundaries. In our networks, no-slip boundary conditions are enforced as soft constraints in the form of loss function while reasonable accuracy is achieved. We believe that this is due to our larger network size enabled by randomly selecting smaller batches for processing from a significantly larger pool of random spatial points (1000 times the number of samples in a single batch). The sampling and batch sample selection are part of the NVIDIA Modulus framework. The soft constraint approach does not perform as well as the hard constraint approach, but hard boundary constraints are difficult to extend to Neumann constraints and implement on complex geometry and may pose difficulty for future scaling up.

As expected, Table 2 results demonstrate that increasing the network width while maintaining the same depth decreases errors significantly but at the same time, increases requirements for GPU memory and computational time. Interestingly, incorporating TSC inputs leads to significant improvements in accuracy and a reduction of computational resources needed. The network incorporating TSC with a width of 256 produces a similar accuracy as the network without TSC with twice the width (512) and takes approximately 50% less time to train. The reduction in time is related to the reduced network size, such that the number of trainable parameters is reduced from 790,531 to 200,707. Further, training converges data shows that with the TSC, losses could converge to be lower, and converge faster than the network without TSC with twice the network size.

Next, using the Mixed Network architecture, we train 16 case geometries and evaluate accuracy on a validation set comprising 45 unseen case geometries, as depicted in Fig. 2 and summarized our findings in Table 3. Again, the network with TSC, having a smaller width of 856, demonstrates statistically comparable accuracy to the network without TSC with an approximately 50% greater width of 1284, despite having more than halved the number of trainable parameters, and reduced training time by approximately 20%.

**Table 3:** Comparison of relative L2 error and computational expense for multi-case training across various a range of narrowing severity, ***A*** and narrowing length, ${\varvec{\upsigma}}$ using various neural network depth sizes, as well as a smaller NN when employing “local coordinates inputs”.

NN size [Layer x Neurons]	No. of hyperparameters	ε_u_ (n = 45)	ε_v_ (n = 45)	ε_p_ (n = 45)	Approx. computational time (min)	GPU usage (GB)
4 × 1070 without TSC	3,443,263	3.2 ± 1.8%*	7.4 ± 2.3%*	11.2 ± 6.3%*	450	4.54
4 × 1284 without TSC	4,956,243	1.4 ± 0.7%	3.9 ± 1.4%	4.4 ± 2.6%	500	5.30
4 × 1712 without TSC	8,806,531	1.2 ± 0.7%*	3.7 ± 1.8%*	3.4 ± 1.9%*	> 700	6.87
4 × 856 with TSC	2,211,907	1.5% ± 0.6%	4.5 ± 1.1%	5.6 ± 2.5%	400	4.18

NN—neural network; LCI—local coordinate Inputs; ε_u_, ε_v_, ε_p_—relative L2 error for U velocity, V velocity and pressure respectively. Error data are presented as mean ± standard deviation. * p < 0.05 compared to “4 × 856 with TSC”.

The superior accuracy provided by TSCs suggests that flow dynamics in tube-like structures are strongly correlated to tube-specific coordinates, and the network does not naturally produce such parameters without deliberate input. Due to these observed advantages, we incorporated TSCs in all further multi-case PINN investigations.

### Comparison of various multi-case PINN architectures

We conducted a comparative analysis to determine the best network architecture for multi-case PINN training for tube flows. We design the networks such that the number of trainable parameters is standardized across the three network architectures for a controlled comparison. Two experiments are conducted, where the trainable parameters are approximately 2.2 million and 0.8 million. The network size parameters are shown in Table 4, while the results are shown in Table 5. We investigated L2 errors for velocities, pressures, and wall shear stresses (WSS).

**Table 4:** Details of NN architecture size and number of hyperparameters.

NN architecture	Main NN size	Secondary hypernetwork size	No. of hyperparameters
Mixed	856, 856, 856, 856	–	2,214,475
Hypernetwork	256, 256, 256, 256	32, 32, 32, 32, 32, 10	2,215,275
Modes	851, 851, 851	32, 32, 32, 32, 32, 10	2,217,282
Downsized Mixed	516, 516, 516, 516	–	808,575
Downsized Hypernetwork	256, 256, 256, 256	32, 32, 32, 32, 32, 3	808,303
Downsized Modes	513, 513, 513	32, 32, 32, 32, 32, 3	807,296

NN—neural network.

**Table 5:** Comparison of relative L2 error and computational expense for multi-case training across various a range of narrowing severity, ***A*** and narrowing length, ${\varvec{\upsigma}}$ using different neural network architectures with approximately 2.2 million hyperparameters for each.

NN architecture	Mixed	Hypernetwork	Modes		Downsized mixed	Downsized hypernetwork	Downsized modes
ε_u_ (n = 45), %	1.5 ± 0.6	0.8 ± 0.4	0.4 ± 0.2		2.1 ± 1.5	0.9 ± 0.4	5.2 ± 3.7
ε_v_ (n = 45), %	4.5 ± 1.1	3.6 ± 0.8	2.1 ± 0.5		6.0 ± 2.4	4.2 ± 1.2	12.4 ± 5.4
ε_p_ (n = 45), %	5.6 ± 2.5	2.3 ± 1.2	1.2 ± 0.5		8.0 ± 6.3	2.2 ± 1.2	13.1 ± 7.5
ε_WSS_mag_ (n = 45), %	1.5 ± 0.7	1.3 ± 0.6	1.0 ± 0.5		1.9 ± 0.8	1.3 ± 0.6	5.4 ± 3.7
Computational Time, mins	500	Above 2000	300		300	Above 2000	150
GPU memory usage, GB	4.18	21.7	3.64		2.66	20.98	2.34

Repeat comparison was done but with “downsized” NN sizes for each architecture, standardized to approximately 0.8 million hyperparameters.

NN—neural network; ε_u_, ε_v_, ε_p_, ε_WSS_mag_—relative L2 error for U velocity, V velocity pressure and wall shear stress magnitude. Error data are presented as mean ± standard deviation. Differences in errors across the three network types are all significant (p < 0.05).

From Table 5, it can be observed that with a larger network size (2.2 million trainable parameters), the Modes network has the lowest relative L2 errors, averaged across all testing cases, of between 0.4 and 2.1%, which is significantly more accurate than the Mixed network and the Hypernetwork. Results indicate that the percentage errors of the spanwise velocity, $v$, are higher than those in the streamwise velocity, $u$, due to the larger amplitude of $u$. As such, errors in WSS are aligned to errors in u rather than v. However, when a smaller network size (0.8 million trainable parameters) is used, the Hypernetwork displayed the highest accuracy, followed by the Mixed network and then the Modes network.

**Figure 5:**
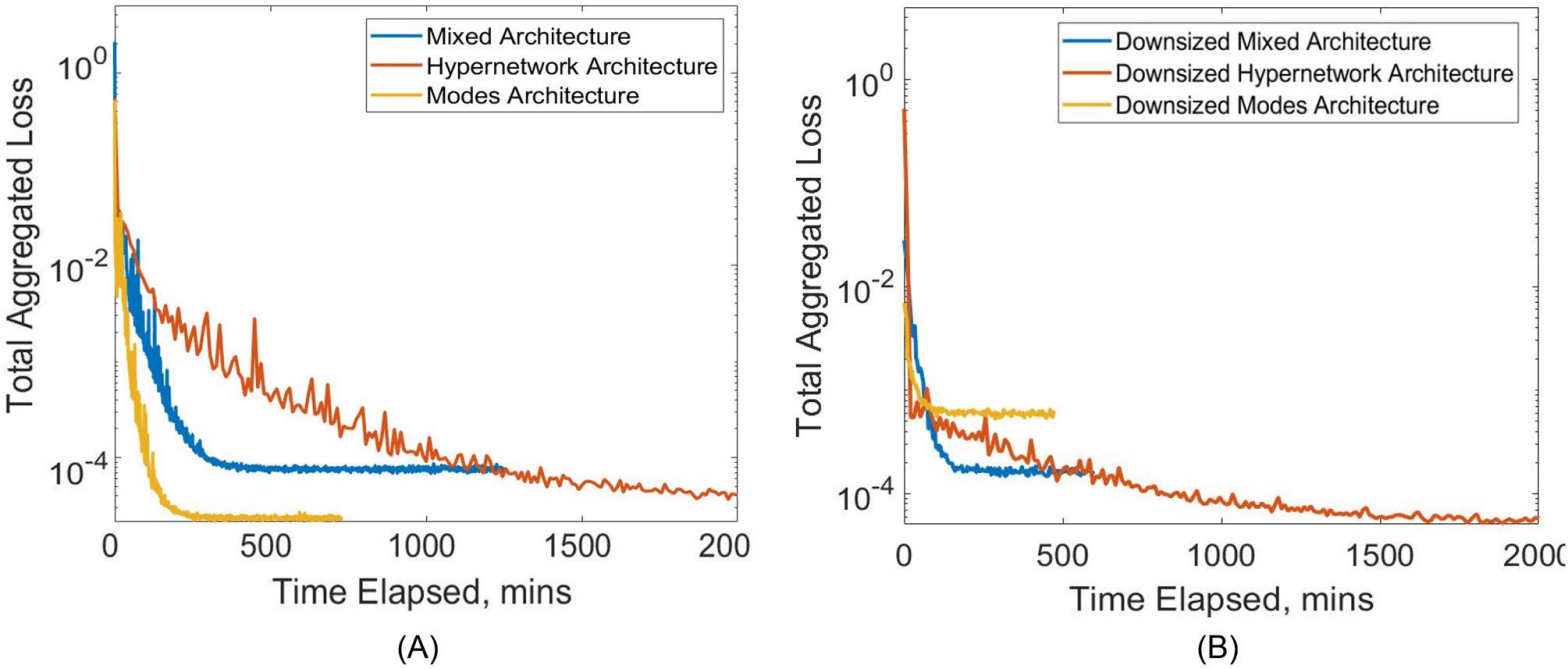
(**A**) Comparison of convergence for total aggregated loss plotted against time taken in minutes for multi-case training across various a range of narrowing severity, ***A*** and narrowing length, ${{\varvec{\upsigma}}}$, between the three different neural network architectures. (**B**) Repeat comparison was done but with smaller NN sizes for each architecture, standardized to approximately 0.8 million hyperparameters, compared to 2.2 million hyperparameters in (**A**). (**A**) Architecture Comparison with Approx. 2.1 million Hyperparameters. (**B**) Architecture Comparison with Approx. 0.8 million Hyperparameters.

These results are also observable in Fig. 5, which illustrates the convergence of loss functions under various network training. Specifically, Fig. 5A demonstrates that the Modes network exhibits the swiftest convergence with the lowest total aggregated loss. This was followed by the Hypernetwork, which has the next lowest converged loss but has a very slow slower convergence rate. The mixed network exhibits the highest converged loss but shows a moderate convergence speed. In contrast, Fig. 5B demonstrates the convergence patterns when there is a smaller number of trainable parameters. Although the order in the speed of convergence remains consistent, the Modes Network now has the highest converged aggregated loss. This highlights the necessity of a sufficiently large network size for the effectiveness of the Modes Network.

Another advantage of the Modes Network is that it takes up the lowest GPU memory and training time (Table 5). Further, although the Hypernetwork was more accurate than the Mixed Network, the training time and GPU memory required was several times that of the Mixed Network. The Hypernetwork consumes at least 13 times more memory than the Modes Network, and several times longer to converge.

**Figure 6:**
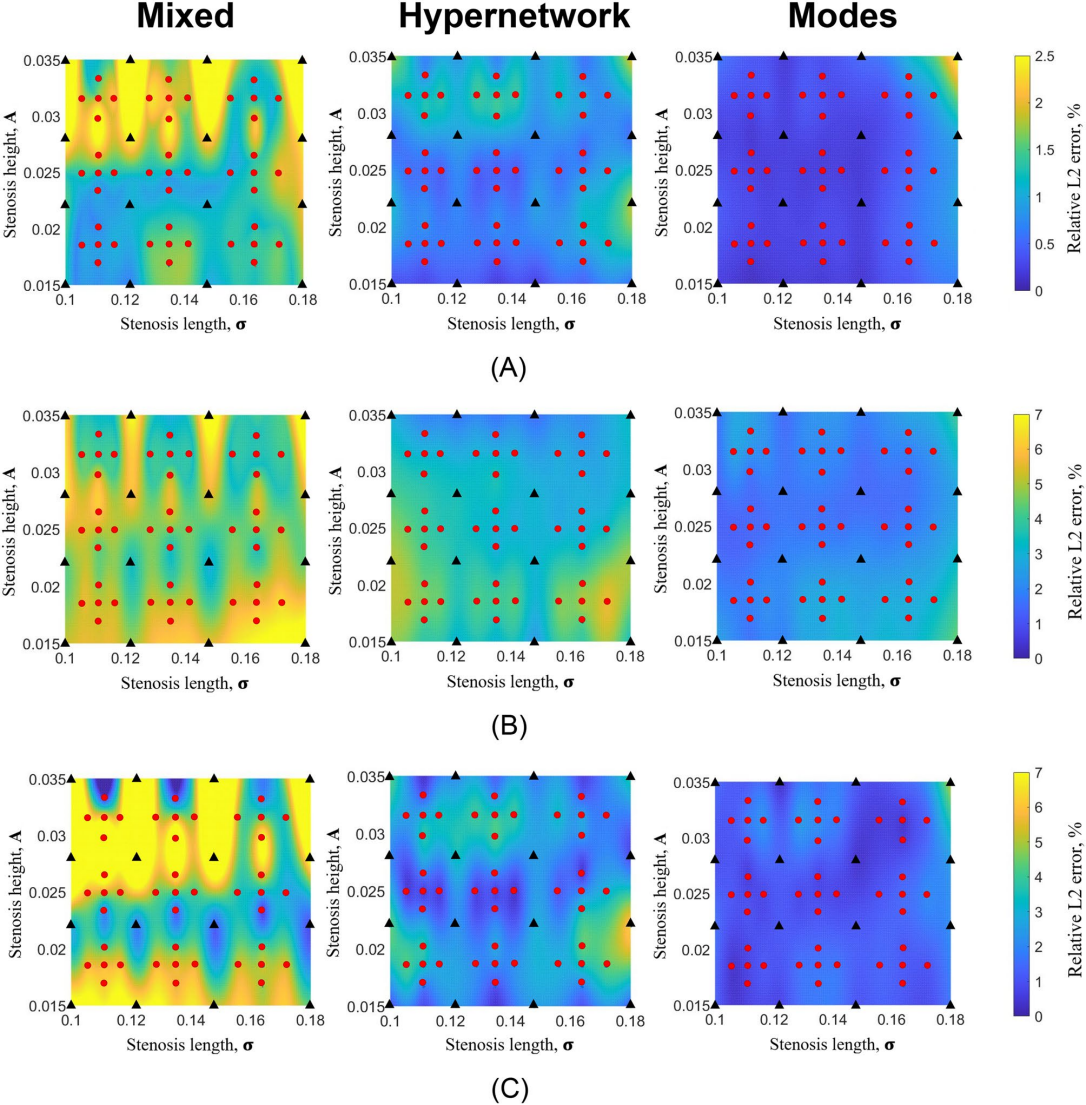
Color contour plot of relative L2 error of (**A**) U velocity, (**B**) V velocity and (**C**) pressure from multi-case training across various range of narrowing severity, ***A*** and narrowing length, ${{\varvec{\upsigma}}}$, between the three different neural network architecture with 2.2 million hyperparameters. (**A**) U relative L2 error. (**B**) V relative L2 error. (**C**) P relative L2 error.

**Figure 7:**
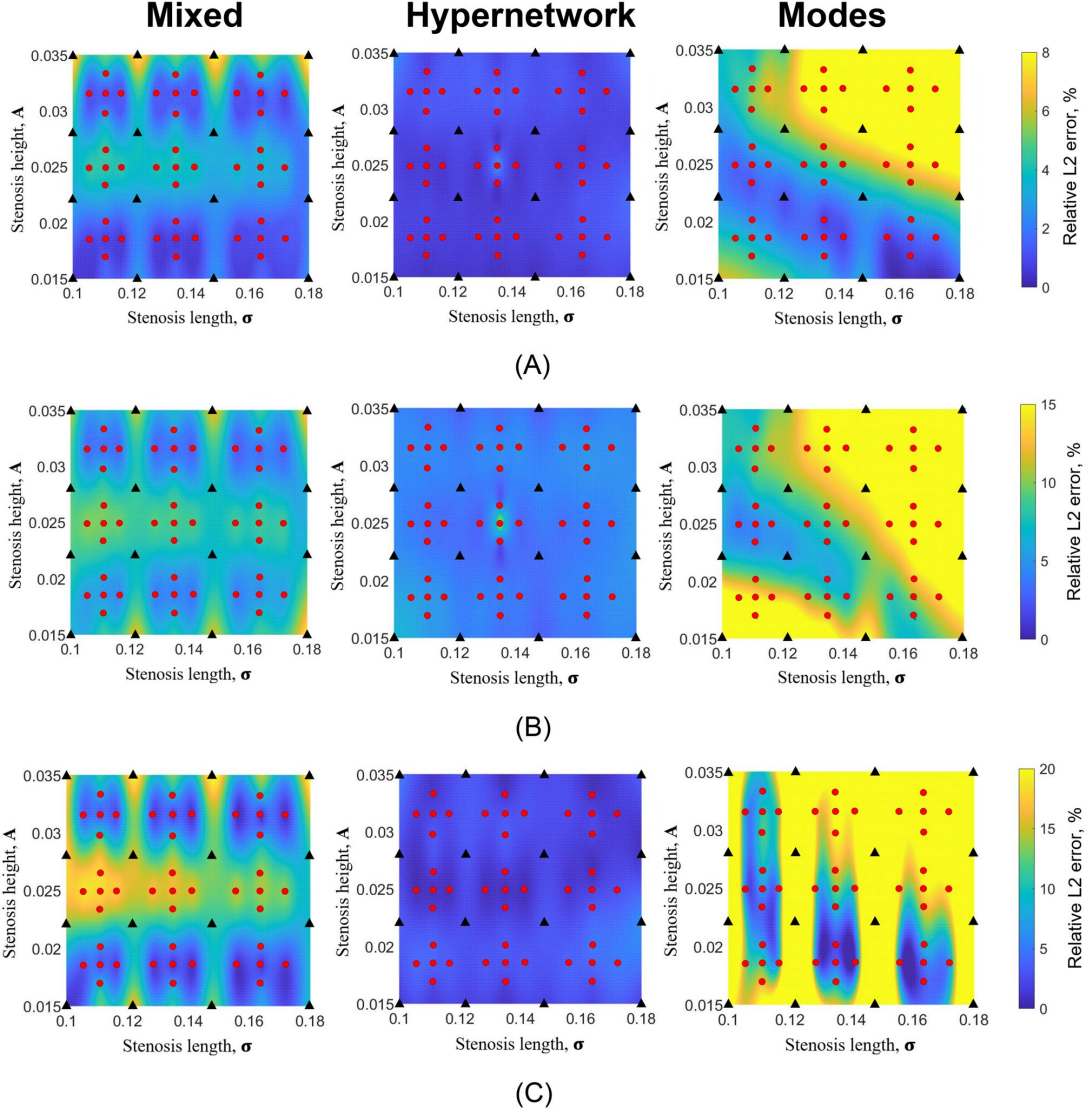
Colour contour plot of relative L2 error plot of (**A**) U velocity, (**B**) V velocity and (**C**) pressure from multi-case training across various range of narrowing severity, ***A*** and narrowing length, ${{\varvec{\upsigma}}}$, between the three different neural network architecture with a reduced number of hyperparameters (0.8 million). (**A**) U relative L2 error. (**B**) V relative L2 error. (**C**) P relative L2 error.

Figures 6 and 7 show the distribution of relative L2 errors across the geometric parameter space for the three networks. Training geometric cases are indicated as black triangles while testing cases are indicated as red dots. It can be observed that the geometric parameter spaces in between training cases have good, low errors similar to errors of training cases, demonstrating that the multi-case PINN approach of training only in some cases is feasible and can ensure accuracy in unseen cases. The results further demonstrate that cases with larger ***A*** parameters tend to have larger errors. This is understandable as larger ***A*** corresponds to more severe narrowing and a flow field with higher spatial gradients.

The results suggest that the Modes network has the potential to be the most effective and efficient network; however, a sufficiently large network size is necessary for accuracy.

### Utilizing gradient-enhanced PINNs (gPINNs)

We test the approach of adding derivatives of governing and boundary equations with respect to case parameters as additional loss functions, and investigate enhancements to accuracy and training efficiency, using the networks with approximately 2.2 million trainable parameters. The networks are trained with the original loss functions until convergence before the new derivative loss function is added and the training restarted.

**Figure 8:**
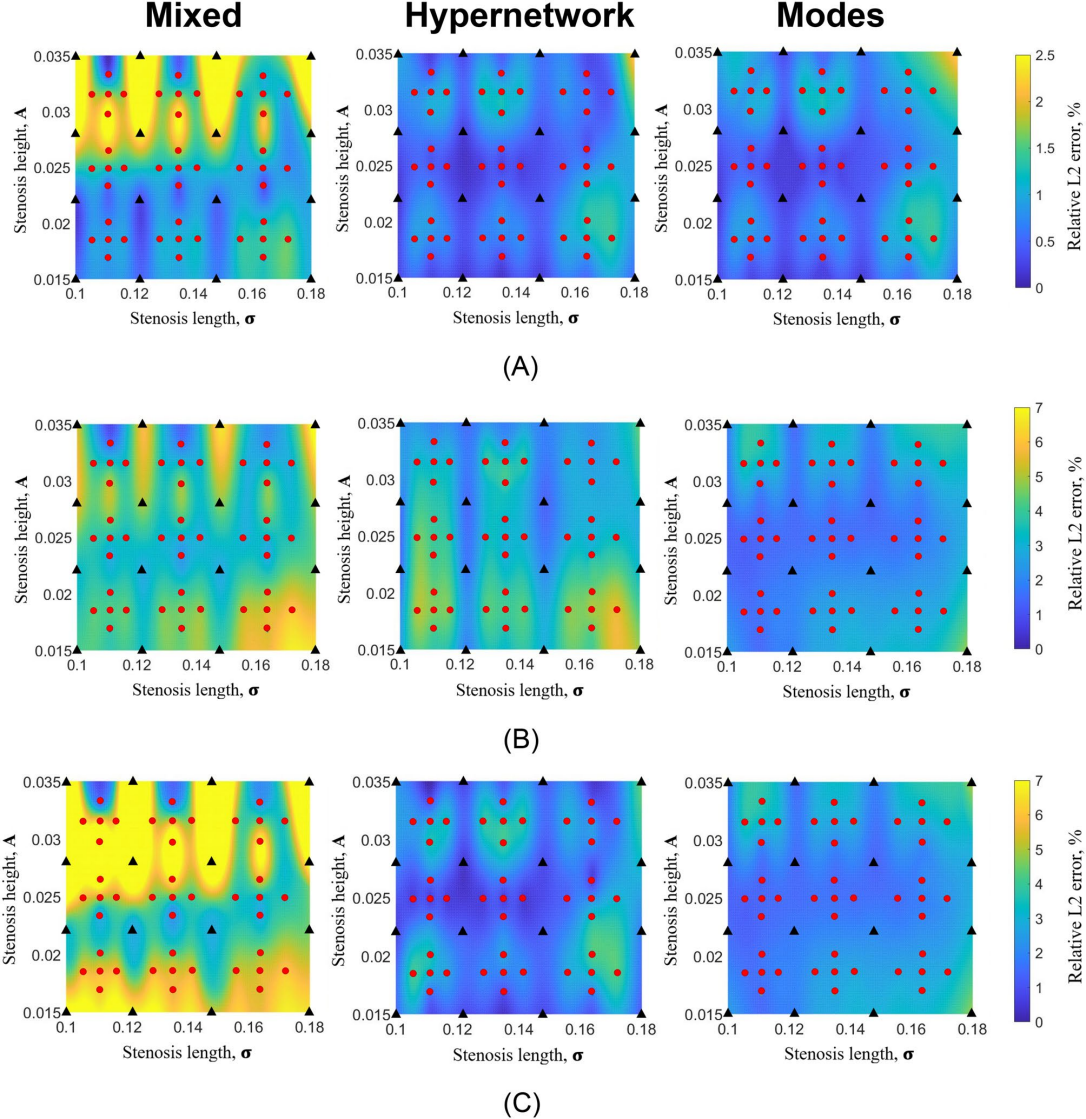
Colour contour plots of relative L2 error plot of (**A**) U velocity, (**B**) V velocity and (**C**) pressure from multi-case training across various ranges of narrowing severity, ***A*** and narrowing length, ${{\varvec{\upsigma}}}$, between the three different neural network architectures with 2.2 million hyperparameters, after adding the derivatives of governing equations and boundary conditions wrt. case parameters as additional loss functions. (**A**) U relative L2 error. (**B**) V relative L2 error. (**C**) P relative L2 error.

**Figure 9:**
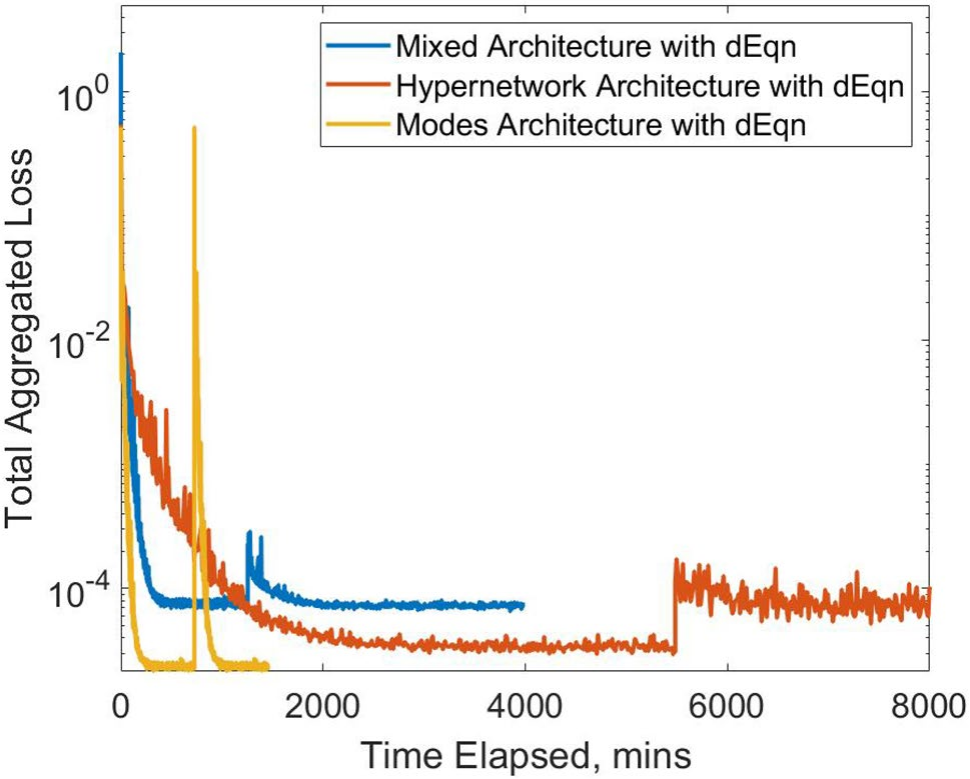
Comparison of convergence for total aggregated loss plotted against the time taken in minutes for multi-case training with approximately 2.2 million hyperparameters for the three different PINN methodologies. The derivative of governing equations and boundary conditions (gPINN) is added as an additional loss term after the initial training is converged, and noticeable spikes in loss are observed.

The convergence plot is illustrated in Fig. 9, while the results are shown in Table 6. Results in Fig. 8 indicate that this approach generally led to small-magnitude improvements in velocities and pressure errors, most of which are statistically significant. Significant improvements are the most evident for the Mixed network, where all output parameters significantly improve. This is followed by the Hypernetwork, where the streamwise velocity, $u$ and pressure errors significantly improve. However, for the Modes network, error reduction is not evident, and the accuracy of the spanwise velocity, $v$ deteriorated. Imposing the additional loss functions causes a roughly double increase in training time and a 3–4 times increase in GPU memory requirements for the Mixed and Modes networks.

**Table 6:** Relative L2 error and computational expense for multi-case training with approximately 2.2 million hyperparameters for each NN architecture with derivative of governing equations and boundary conditions, gPINN, as an additional loss term.

NN architecture	Mixed	Hypernetwork	Modes
ε_u_ (n = 45), %	1.4 ± 0.5*	0.7 ± 0.3^†^	0.4 ± 0.2
ε_v_ (n = 45), %	4.2 ± 1.1*	3.5 ± 0.8	2.5 ± 0.8^‡^
ε_p_ (n = 45), %	5.1 ± 2.1*	2.2 ± 1.2^†^	1.2 ± 0.4
ε_WSS_mag_ (n = 45), %	1.5 ± 0.6*	1.2 ± 0.6^†^	1.0 ± 0.5
Computational Time, mins	1000	Above 3500	600
GPU memory usage, GB	13.5	21.9	12.2

^*,**†**,**‡**^P < 0.05 when comparing each respective NN architecture with and without additional derivative loss terms (gPINN). NN—neural network; ε_u_, ε_v_, ε_p_, ε_WSS_mag_—relative L2 error for U velocity, V velocity, pressure and wall shear stress magnitude. Error data are presented as mean ± standard deviation.

In summary, the derivatives loss function yielded improvements for the Mixed Network and Hypernetwork but did not show improvements for the Modes Network.

## Discussion

In this study, we investigated three common training strategies for multi-case PINN applied to fluid flows in tube-like structures. Additionally, we investigated the use of gPINN and TSC to enhance these networks. While our algorithms are not ready for biomedical applications, they lay the groundwork for future work in scaling up to 3D complex geometries with more clinically relevant flows. If successful, this approach could offer substantial advantages over the traditional CFD approach.

Traditional CFD simulations are required for every new vascular or airway geometry encountered, and even though this is currently a well-optimized and efficient process, a minimum of several tens of minutes is required for meshing and simulating each case. Much of this simulation process is repetitive, such as when very similar geometries are encountered, but the same full simulation is required for each of such cases and transfer learning is not possible without machine learning. In contrast, multi-case PINN enables a single learning process for a range of geometries, avoiding redundant computations and potentially providing real-time results. Real-time capabilities could encourage clinical adoption, enhance clinical decision-making, and facilitate faster engineering computations, ultimately contributing to increased result sample sizes for demonstrating the clinical impact of biomechanical factors.

Similar to previous investigations^9,10^, one key motivation for adopting multi-case PINN is its ability to pre-train on a small series of cases, allowing real-time results for unseen cases close to the trained cases. In the original form, PINN is case-specific, the training time required for single cases far exceeds that required for traditional CFD simulations, for results with similar accuracy^20^. There is thus no reason for using PINN to solve such single cases, unless inverse computing, such as matching certain observations in the flow field is required^21^. At present, using our trained PINN to solve 2D tube flows only yields small advantages compared to conventional CFD of steady 2D flows, but in future when the 3D version of the multi-case PINN is available, this advantage can become more pronounced.

When comparing the Mixed, Modes and Hypernetworks, we designed our study to utilize various extents of hyperparameter networks, with the Hypernetwork approach representing the fullest extent, the Mixed network representing the minimum extent, and the Modes network falling in between. Results show that the hypernetwork can yield better results than the Mixed network when the number of trainable parameters for both networks is retained. This agrees with previous investigations on the Hypernetwork approach, where investigators found that a reduced network size to achieve the same accuracy is possible^7,8^. However, the hypernetwork approach requires a large GPU memory, because the links between the hyperparameter network and the first few layers of the main PINN network result in a very deep network with many sequential layers, and the backward differentiation process via chain rule requires the storage of many more parameters. The complexity of this network architecture also resulted in long training times and slower convergence.

In comparison, the Modes Network reduces complexity, resulting in faster training times and faster convergence. This approach aligns with the “sparse hypernetwork” approach, where the hypernetwork supplies only a subset of the weights in the main network, which corroborates our observations of significantly reduced memory and computational requirements without sacrificing performance^11^. The good performance of the Modes Network suggests that the complexity in the Hypernetwork is excessive and is not needed to achieve the correct flow fields. The Modes network also has similarities to reduced order PINNs, such as proposed by Buoso et al.^22^ for simulations of cardiac myocardial biomechanics. Buoso et al. use shape modes for inputs into the PINN and utilize outputs as weights for a set of motion modes, where all modes are pre-determined from statistical analysis of multiple traditional simulations. Our Modes Network similarly calculates a set of modes, ${\varvec{q}}$ in Eq. (14), and used PINN outputs as weights for these modes to obtain flow field results. The difference, however, is that we determined these modes from the training itself, instead of pre-determining them through traditional CFD simulations.

Another important result here is the improved accuracy provided by tube-specific coordinate inputs when simulating tube flows. This not only accelerates convergence rates and reduces computational costs, but it also leads to improved accuracies as well. An explanation for this is that the tube flow fields have a strong correlation to the tube geometry and thus tube-specific coordinate inputs, and having such coordinates directly input into the PINN allows it to find the solution more easily. For example, in laminar tubular flow, flow profiles are likely to approximate the parabolic flow profile, which is a square function of the y-coordinates, and as such multiplicative expressions are needed for the solution. By itself, the fully connected network can approximate squares and cross-multiplication of inputs, but this requires substantial complexity and is associated with approximation errors. Pre-computing these second-order terms for inputs into the network can reduce the modelling burden and approximation errors, thus leading to improved performance with smaller networks. The strategy is likely not limited to tube flows, for any non-tubular flow geometry, coordinate parameters relevant to that geometry are likely to improve PINN performance as well. In our experiments with the simple parameterized 2D narrowing geometries, tube-specific coordinates can be easily calculated, however, for more complex tube geometries, specific strategies to calculate these coordinates are needed. Such computations will likely need to be in the form of an additional neural network because derivates of the coordinates will need to be computed in the multi-case PINN architecture.

Our investigation of the gPINN framework featuring the gradient of the loss functions shows its usefulness for the Mixed Network and Hypernetwork but not the Modes Network. In the Hypernetwork and Mixed network, this gPINN modifies the solution map, reducing loss residuals for cases close to the trained cases by enforcing a low gradient of loss across case parameters. In the Modes Network, solutions are modelled in reduced order, as they are expressed as a linear finite combination of solution modes, and consequently already exhibit smoothness across case parameters. This is thus a possible explanation for why the Modes Network does not respond to the gPINN strategy. Further, the Modes Network shows an excessive increase in losses when the loss function derivatives were added during the training (Fig. 9), which may indicate an incompatibility of the reduced order nature of the network with the gPINN framework, where there are excessive changes to the solution map in response to adjusting this new loss function.

## Conclusion

The results suggest the feasibility of employing unsupervised PINN training through the multi-PINN approach to generate real-time fluid dynamics results with reasonable accuracies compared to CFD results. Our findings suggest that the most effective strategy for multi-case PINN in tube-like structures is the Modes Network, particularly when combined with tube-specific coordinate inputs. This approach not only provides the best accuracy but also requires the least computational time and resources for training. It is important to note that our investigations are confined to time-independent 2D flows within a specific geometric parameter space of straight, symmetric channels without curvature and a limited range of Reynolds numbers. Despite these limitations, our results may serve as a foundation for future endeavors, scaling up to 3D simulations with time variability and exploring a broader spectrum of geometrical variation.

## References

[1] Pijls, N. H. J. *et al.* Measurement of fractional flow reserve to assess the functional severity of coronary-artery stenoses. *N. Engl. J. Med.* **334**, 1703–1708. 10.1056/nejm199606273342604 (1996). 8637515 10.1056/NEJM199606273342604

[2] Bordones, A. D. *et al.* Computational fluid dynamics modeling of the human pulmonary arteries with experimental validation. *Ann. Biomed. Eng.* **46**, 1309–1324. 10.1007/s10439-018-2047-1 (2018). 29786774 10.1007/s10439-018-2047-1 PMC6095803

[3] Zhou, M. *et al.* Wall shear stress and its role in atherosclerosis. *Front. Cardiovasc. Med.* **10**, 1083547 (2023). 37077735 10.3389/fcvm.2023.1083547 PMC10106633

[4] Frieberg, P.A.-O. *et al.* Computational fluid dynamics support for fontan planning in minutes, not hours: The next step in clinical pre-interventional simulations. *J. Cardiovasc. Transl. Res.* **15**(4), 708–720 (2022). 34961904 10.1007/s12265-021-10198-6 PMC9622535

[5] Raissi, M., Perdikaris, P. & Karniadakis, G. E. Physics-informed neural networks: A deep learning framework for solving forward and inverse problems involving nonlinear partial differential equations. *J. Comput. Phys.* **378**, 686–707. 10.1016/j.jcp.2018.10.045 (2019).

[6] Kashefi, A. & Mukerji, T. Physics-informed PointNet: A deep learning solver for steady-state incompressible flows and thermal fields on multiple sets of irregular geometries. *J. Comput. Phys.* **468**, 111510. 10.1016/j.jcp.2022.111510 (2022).

[7] Ha, D., Dai, A. & Quoc. HyperNetworks. arxiv:1609.09106 (2016).

[8] Filipe, Chen, Y.-f. & Sha, F. *HyperPINN: Learning Parameterized Differential Equations with Physics-Informed Hypernetworks* (Springer, 2021).

[9] Sun, L., Gao, H., Pan, S. & Wang, J.-X. Surrogate modeling for fluid flows based on physics-constrained deep learning without simulation data. *Comput. Methods Appl. Mech. Eng.* **361**, 112732 (2019).

[10] Oldenburg, J., Borowski, F., Öner, A., Schmitz, K.-P. & Stiehm, M. Geometry aware physics informed neural network surrogate for solving Navier-Stokes equation (GAPINN). *Adv. Model. Simul. Eng. Sci.* **9**, 8. 10.1186/s40323-022-00221-z (2022).

[11] Shazeer, N. *et al.**Outrageously Large Neural Networks: The Sparsely-Gated Mixture-of-Experts Layer* (Springer, 2017).

[12] Lu, L., Jin, P., Pang, G., Zhang, Z. & Karniadakis, G. E. Learning nonlinear operators via DeepONet based on the universal approximation theorem of operators. *Nat. Mach. Intell.* **3**, 218–229. 10.1038/s42256-021-00302-5 (2021).

[13] Wang, S., Wang, H. & Perdikaris, P. Learning the solution operator of parametric partial differential equations with physics-informed DeepONets. *Sci. Adv.* **7**, eabi8605. 10.1126/sciadv.abi8605 (2021). 34586842 10.1126/sciadv.abi8605 PMC8480920

[14] Yu, J., Lu, L., Meng, X. & Karniadakis, G. E. Gradient-enhanced physics-informed neural networks for forward and inverse PDE problems. *Comput. Methods Appl. Mech. Eng.* **393**, 114823. 10.1016/j.cma.2022.114823 (2022).

[15] Diederik, H. & Ba, J. *Adam: A Method for Stochastic Optimization* (Springer, 2017).

[16] Atilim, B. & Alexey, J. *Automatic Differentiation in Machine Learning: A Survey* (Springer, 2018).

[17] Hennigh, O. *et al.* 447–461 (Springer International Publishing, 2021).

[18] Jin, X., Cai, S., Li, H. & Karniadakis, G. E. NSFnets (Navier-Stokes flow nets): Physics-informed neural networks for the incompressible Navier-Stokes equations. *J. Comput. Phys.* **426**, 109951. 10.1016/j.jcp.2020.109951 (2021).

[19] Jagtap, A. D., Kawaguchi, K. & Karniadakis, G. E. Adaptive activation functions accelerate convergence in deep and physics-informed neural networks. *J. Comput. Phys.* **404**, 109136. 10.1016/j.jcp.2019.109136 (2020). 10.1098/rspa.2020.0334 PMC7426042 32831616

[20] Moser, P., Fenz, W., Thumfart, S., Ganitzer, I. & Giretzlehner, M. Modeling of 3D blood flows with physics-informed neural networks: Comparison of network architectures. *Fluids* **8**, 46. 10.3390/fluids8020046 (2023).

[21] Arzani, A., Wang, J.-X. & D’Souza, R. M. Uncovering near-wall blood flow from sparse data with physics-informed neural networks. *Phys. Fluids* **33**, 7 (2021).

[22] Buoso, S., Joyce, T. & Kozerke, S. Personalising left-ventricular biophysical models of the heart using parametric physics-informed neural networks. *Med. Image Anal.* **71**, 102066. 10.1016/j.media.2021.102066 (2021). 33951597 10.1016/j.media.2021.102066

